# Dampening Spontaneous Activity Improves the Light Sensitivity and Spatial Acuity of Optogenetic Retinal Prosthetic Responses

**DOI:** 10.1038/srep33565

**Published:** 2016-09-21

**Authors:** John Martin Barrett, Gerrit Hilgen, Evelyne Sernagor

**Affiliations:** 1 Institute of Neuroscience, Faculty of Medical Sciences, Newcastle University, Framlington Place, Newcastle-upon-Tyne, NE2 4HH, United Kingdom

## Abstract

Retinitis pigmentosa is a progressive retinal dystrophy that causes irreversible visual impairment and blindness. Retinal prostheses currently represent the only clinically available vision-restoring treatment, but the quality of vision returned remains poor. Recently, it has been suggested that the pathological spontaneous hyperactivity present in dystrophic retinas may contribute to the poor quality of vision returned by retinal prosthetics by reducing the signal-to-noise ratio of prosthetic responses. Here, we investigated to what extent blocking this hyperactivity can improve optogenetic retinal prosthetic responses. We recorded activity from channelrhodopsin-expressing retinal ganglion cells in retinal wholemounts in a mouse model of retinitis pigmentosa. Sophisticated stimuli, inspired by those used in clinical visual assessment, were used to assess light sensitivity, contrast sensitivity and spatial acuity of optogenetic responses; in all cases these were improved after blocking spontaneous hyperactivity using meclofenamic acid, a gap junction blocker. Our results suggest that this approach significantly improves the quality of vision returned by retinal prosthetics, paving the way to novel clinical applications. Moreover, the improvements in sensitivity achieved by blocking spontaneous hyperactivity may extend the dynamic range of optogenetic retinal prostheses, allowing them to be used at lower light intensities such as those encountered in everyday life.

Retinitis pigmentosa (RP) is a progressive retinal dystrophy in which initial death of rod photoreceptors, followed by slower cone death, causes visual impairment and eventual blindness. It has a worldwide prevalence of approximately 1 in 3–4000^1^,^2^. Presently, the only clinically available vision-restoring treatment for RP is retinal prosthesis, in which an array of electrodes and/or photodiodes is implanted into the eye in order to stimulate surviving inner retinal cells^3^,^4^,^5^. Such devices have been reasonably successful, with a number of patients experiencing improvements on various visual tasks and in activities of daily living^3^,^4^ that persist for years after initial implantation^3^. Nevertheless, the returned vision remains relatively poor, with the best reported Snellen visual acuity of any retinal prosthesis patient being 20/546^4^, still within the range of legal blindness (usually defined as 20/200).

To overcome this, numerous groups are currently investigating optogenetic approaches to retinal prosthesis, in which light-sensitive proteins are transgenically expressed in surviving inner retinal cells^6^,^7^,^8^,^9^. Such approaches have several advantages over electrical prosthesis, for example they do not require a permanent implant, stimulation can be targeted to specific cell types or subtypes, and they can provide inhibitory as well as excitatory stimulation^6^. However, an often overlooked aspect of photoreceptor dystrophies is the inner retinal remodeling that takes place^10^,^11^,^12^ following photoreceptor death, leading to pathological retinal hyperactivity in the form of low-frequency local field potential oscillations and rhythmic bursting of retinal ganglion cells (RGCs)^13^,^14^,^15^. Recently, our group^16^ and others^17^,^18^ have shown that blocking this activity improves the signal-to-noise ratio (SNR) of RGC responses to residual photoreceptor, electrical, or optogenetic stimulation, and that gap-junction blockade – either using meclofenamic acid (MFA)^16^,^17^ or genetic knock-out^18^ – is the best way of achieving this.

The aforementioned studies only considered responses to very simple stimuli, such as simple light flashes, electrical microstimulation, or moving bars, and assessed improvements in prosthetic responses information theoretically. Here, we ask whether these results extend to other stimulus classes and to measures of visual performance more relevant to clinical visual assessment. In particular, we asked whether dampening spontaneous activity can improve the light sensitivity, contrast sensitivity, and spatial acuity of optogenetically-evoked RGC population responses. Moreover, we tested this using a large-scale, high-density multielectrode array (MEA), the Biocam Active Pixel Sensor^19^ (APS; 3Brain GmbH, Landquart, Switzerland), which has 4096 electrodes over an active area of 2.67 mm × 2.67 mm and incorporates a standard DLP projector to provide patterned light stimulation. The advantages of this are two-fold: we were able to record hundreds of optogenetically sensitive RGCs per experiment at pan-retinal level, and we were able to present stimuli of arbitrary spatiotemporal complexity over a range of luminances.

## Results

**Figure 1:**
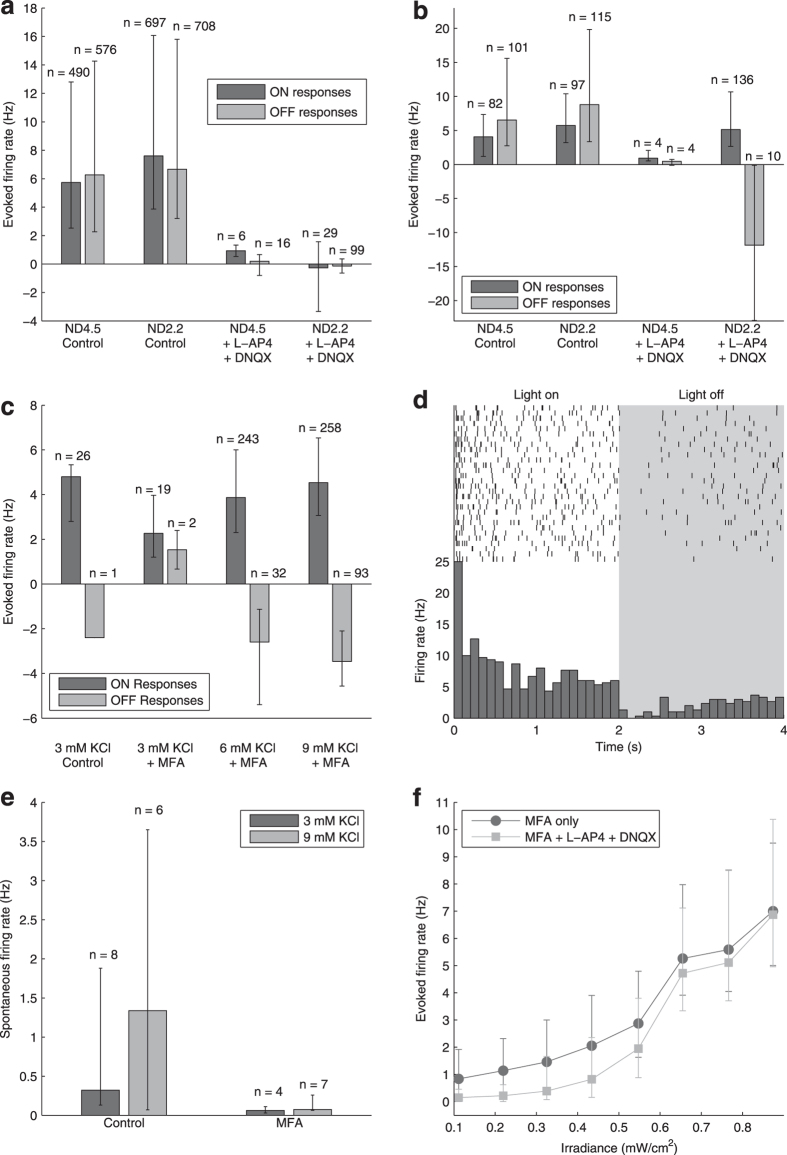
Demonstration of successful optogenetic stimulation with a standard projector. **(a)** Median peak evoked firing rate in the first 250 ms following a 2 s white (dark bars) or black (light grey bars) full field flash for a P52 wild-type mouse retina in control conditions or in the presence of 20 μM DNQX and L-AP4 at two different light intensities. Error bars are inter-quartile range (IQR). *X*-axis units are decadic log units, e.g. 4.5 means the light was attenuated roughly 31000-fold. *n* numbers correspond to the number of responsive cells in each condition. **(b)** As (**a**), but for a P95 *ChR2rd1 hetero* mouse. **(c)** As (**a,b**), but for a P96 *ChR2rd1* mouse at ND2.2, with varying extracellular potassium concentrations and in the absence or presence of 40 μM MFA. All recordings from this retina were performed at ND2.2. **(d)** Raster plot and PSTH for an example RGC recorded from a P96 *ChR2rd1* mouse retina in response to 2s alternating white (white background) and black (light grey background) full fields in the presence of 40 μM MFA with 9 mM KCl. **(e)** Median spontaneous firing rate (recorded in the interval from the beginning of each recording to the first stimulus) on every channel with recorded units in the presence of 3 and 9 mM KCL and in control conditions or with 40 μM MFA. *n* numbers correspond to the number of retinas. **(d)** Median SNR of responses to 250 ms full-fields for all responsive cells as a function of irradiance in a P374 *ChR2rd1* retina in the presence of 40 μM MFA and the absence (•) and presence (■) of 20 μM L-AP4 and DNQX.

All experiments in this study were performed using *rd1* mice. They express a naturally-occurring mutant form of the rod phosphodiesterase gene and experience rapid rod death followed by cone death, with virtually no surviving photoreceptors by P36^20^, and thus serve as a mouse model for RP. *Rd1* mice were crossbred with *ChR2* mice, which express the light-sensitive cation channel channelrhodopsin2^21^ (ChR2) in *Thy1*-expressing cells in the central nervous system, including RGCs^22^,^23^. ChR2-expressing RGCs are photo-activated to firing threshold by blue light^21^,^24^. Crossing *rd1* and *ChR2* mice for at least two generations (the *rd1* mutation is recessive) creates *ChR2rd1* mice. In this study, we used *ChR2rd1* mice of both sexes aged P95–386 and, aside from the data presented in Fig. 1, all mice were aged at least P209, well above the P168 cut-off for cone-mediated light responses previously reported in *rd1* mice^25^. Details of all stimuli are described in the main text and also in the Supplementary Methods.

### Successful Optogenetic Stimulation Using A Standard Projector

First, it was necessary to confirm that our stimulation hardware and conditions are bright enough to stimulate ChR2 without evoking unmanageable stimulation artefacts due to the silicon basis of the APS chip. To do this, we recorded responses to two-second full-field white flashes presented at 0.25 Hz to the retina of mice that had ChR2-expressing RGCs and were heterozygous for the *rd1* mutation^20^ (*ChR2rd1 hetero*). As the *rd1* mutation is recessive, *ChR2rd1 hetero* mice have functional photoreceptors and no retinal degeneration. We recorded responses to 30 flashes each at two light intensities – ND4.5 or 4.4 μW/cm^2^ and ND2.2 or 0.87 mW/cm^2^ – in control conditions and in the presence of 20 μM DNQX and 20 μM L-AP4 to block the glutamatergic pathway from photoreceptors to RGCs. First, as a control, Fig. 1a shows the results of this experiment for a P52 wild-type mouse, i.e. with no ChR2-expressing RGCs and no *rd1* phenotype. We used a bootstrap test to detect responsive cells (Supplementary Methods) and compared the change in firing rate before and after the transition from dark to light and vice-versa. In control conditions, the retina showed strong ON and OFF responses to bright and dim light, but in the presence of the glutamatergic blockers, no light responses remained. When we repeated this experiment with a *ChR2rd1 hetero* mouse, ON responses to bright light persisted after glutamatergic blockade (Fig. 1b). All other types of light responses were blocked, apart from a handful of cells with decreased firing when the light was turned off. Thus we confirmed that we could stimulate ChR2-expressing RGCs with our system.

Next, we tested whether we could stimulate ChR2-expressing RGCs in retinas from *rd1* homozygous mice (*ChR2rd1* mice). We recorded responses to the same stimulus protocol at ND2.2 in a P96 *ChR2rd1* retina, which is sufficiently old that virtually all photoreceptors should have degenerated^20^,^26^. Figure 1c shows the results of this stimulus protocol in control conditions and in the presence of 40 μM MFA (Sigma Aldrich, St Louis, USA; we wait one hour before recording for the drug to take effect). At normal potassium concentrations (3 mM KCl), responses were very weak and sparse, both in control conditions and after applying MFA, suggesting higher stimulation thresholds in *ChR2rd1* mice compared to *ChR2rd1 hetero* mice – something we have noted with other stimulation systems (John Barrett, unpublished observations). To boost membrane excitability in the hope of obtaining more responses, we increased the potassium concentration to 6 and then 9 mM, yielding stronger and more numerous ChR2 responses (see Fig. 1d for a raster plot and post-stimulus time histogram (PSTH) of one example cell), at the expense of higher spontaneous firing (Fig. 1e, Supplementary Fig. S1). Consistent with ChR2 activation, the only type of OFF response observed at these higher concentrations was a decrease in firing when the light was turned off. Thus, white light at these intensities is capable of evoking ChR2 responses without causing substantial artefacts on the silicon MEA chip. However, the trade-off for acquiring large numbers of robust responses requires raising the extracellular potassium concentration (Fig. 1c, Supplementary Fig. S2). Hence, for all subsequent experiments we raised the potassium concentration of the aCSF to 9 mM before taking any recordings.

As a final control to ensure we were stimulating ChR2 and not surviving cones, we presented one *ChR2rd1* retina with 250 ms full-field stimuli of varying irradiance in the presence of 9 mM KCl and 40 μM MFA before and after applying 20 μM L-AP4 and DNQX. Figure 1f shows the evoked firing rate in response to these stimuli. No change in strength of responses was observed, confirming that the responses were ChR2-mediated and not originating from surviving cones.

### Blockade of Spontaneous Activity Improves Contrast Sensitivity

**Figure 2:**
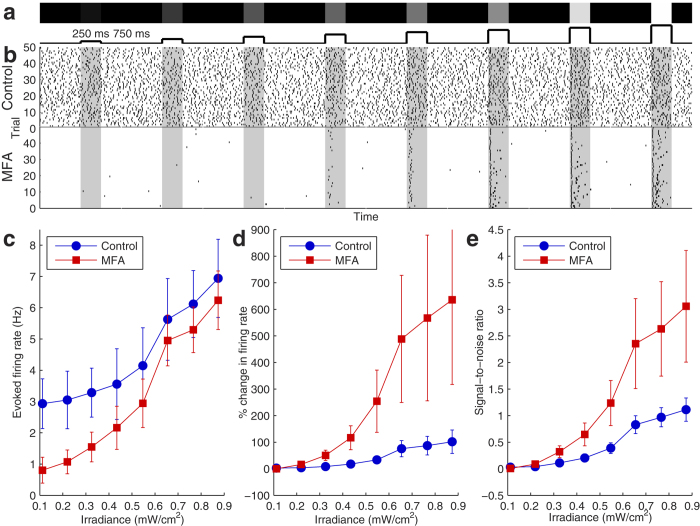
Responses of *ChR2rd1* RGCs to stimulation at different irradiances. **(a)** Stimulus protocol. The retina was presented with 250 ms full-field stimuli at eight intensities (shown graphically in the top row and as a time versus irradiance curve below), one per second. Stimuli are presented in randomized blocks, but each block has been reordered here to show responses in order of increasing irradiance. **(b)** A raster plot from an example cell in control conditions (top row) and after applying 40 μM MFA (bottom row). The grey background indicates when the stimulus shown above in (**a**) was presented. **(c)** Increase in firing during stimulus presentation averaged over all responsive cells in each of *n* = 5 retinas, in control conditions (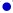) and after applying 40 μM MFA (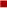), as a function of irradiance. Error bars are mean ± one standard deviation (mean ± s.d.). Two-way repeated measures ANOVA showed significant main effects of irradiance (*F*(7, 28) = 21.57, *p* = 1.0 × 10^−9^) and MFA (*F*(1, 4) = 15.73, *p* = 0.017), as well as a significant interaction (*F*(7, 28) = 12.31, *p* = 4.4 × 10^−7^). **(d)** The data from (**c**) expressed as a percentage of spontaneous activity. The main effects of irradiance (*F*(7, 28) = 26.76, *p* = 8.5 × 10^−11^) and MFA (*F*(1, 4) = 18.15, *p* = 0.013) were significant, as was the interaction (*F*(7, 28) = 14.24, *p* = 9.9 × 10^−8^). **(d)** As (**c,d**) for SNR. The main effects of irradiance (*F*(7, 28) = 62.42, *p* = 2.1 × 10^−15^) and drug (*F*(1, 4) = 24.83, *p* = 0.008), as well as their interaction (*F*(7, 28) = 17.71, *p* = 9.6 × 10^−9^), were all significant.

To investigate how reducing *rd1* spontaneous activity affects responses to ChR2 stimulation over a range of luminances and contrasts, we presented *ChR2rd1* retinas with 250 ms duration full-field stimuli at a rate of 1 Hz and eight different gray levels spanning the full range of the projector output (Fig. 2a). Raster plots of an example ChR2 RGC’s responses to these stimuli are shown in Fig. 2b. Note that, for visualization purposes, in this figure each block of trials has been reordered so that responses to each full field appear in ascending order of luminance. In reality, the stimuli were presented in random order in each block. In control conditions (Fig. 2b, top row) it was difficult to discern any response over the high baseline firing. After blocking the spontaneous activity with MFA (Fig. 2b, bottom row), it was easy to see when the cell responded to the stimulus, with increasing strength as the irradiance increases. In terms of number spikes evoked each stimulus, the responses were slightly but significantly weaker after applying MFA (Fig. 2c).

However, weaker responses may nevertheless be easier to distinguish against reduced spontaneous firing, so we calculated the percentage change in firing compared to baseline and the SNR of the responses. The response period was taken as the entire presentation of the stimulus (250 ms) and blank trials interleaved with the full-field stimuli were used as the baseline. To estimate the standard deviation of the baseline for calculating SNR, the spontaneous firing was binned at 250 ms, to match the length of the response window. Applying MFA significantly increased the evoked firing rate as a percentage of spontaneous activity (Fig. 2d) and the SNR (Fig. 2e), both overall and after controlling for the effect of irradiance.

**Figure 3:**
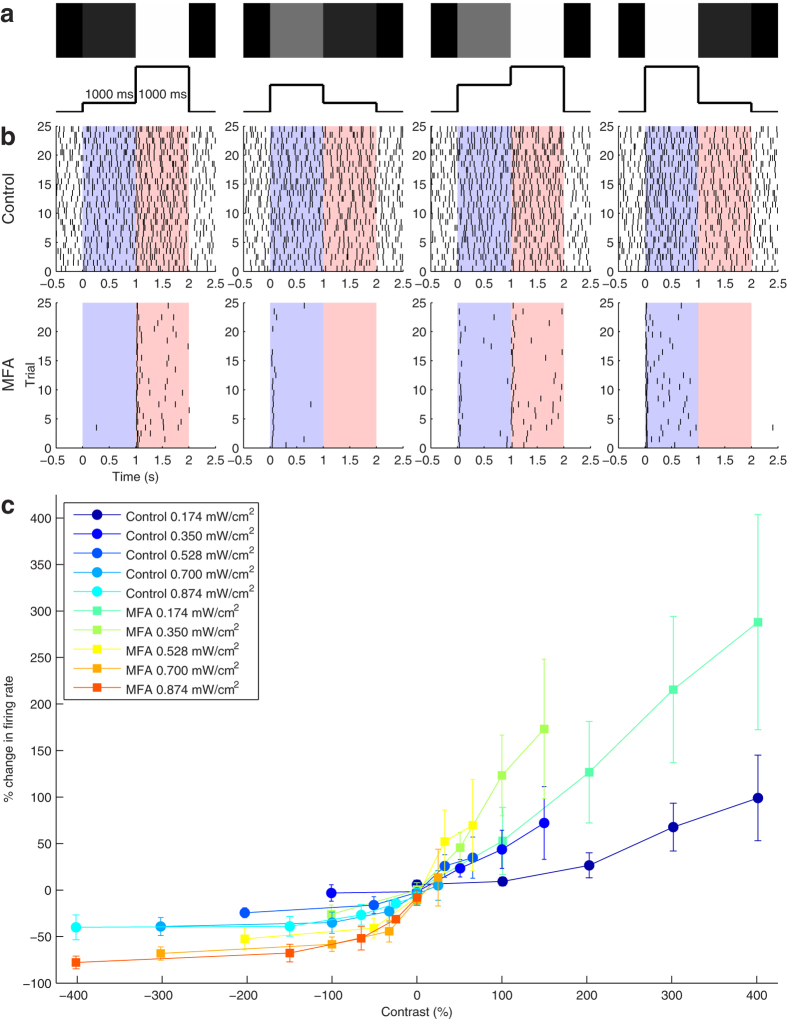
Responses of *ChR2rd1* RGCs to varying contrasts. **(a)** Stimulation protocol. On each trial, a full field of a given irradiance was shown for one second, followed by another full-field of the same or different irradiance for one second, followed by one second of darkness. Four example stimulus pairs are shown graphically in the top row and as a time versus irradiance curve below. **(b)** Raster plots of an example RGC responding to the stimulus pairs shown in (**a**) in control conditions (top row) and after applying 40 μM MFA. The light blue background indicates when the first stimulus was presented and the light red background the second. **(c)** Percentage change in firing between the end of the first stimulus and beginning of the second, averaged over all responsive cells from each of *n* = 5 retinas, in control conditions (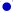) and after applying 40 μM MFA (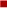), as a function of contrast. Data points have been grouped by the irradiance of the first stimulus of the pair, indicated by the colour of the curve. Error bars are mean ± s.d. Three-way repeated measures ANOVA showed that the main effects of first stimulus irradiance, Weber contrast, and MFA were all significant, as were all two-way interactions, but not the three-way interaction; see Table 1 for details.

To assess how spontaneous hyperactivity affects contrast sensitivity, we used a protocol in which we presented the retina with a full-field stimulus of a certain irradiance for one second, allowing each cell time to adapt to this light level, then immediately presented a new full-field stimulus of a different irradiance (also for one second). Finally, we presented one second of darkness between each pair (Fig. 3a) to allow the ChR2 channels time to recover. Five distinct grey levels were used, for a total of 5 × 5 = 25 contrasts, including 5 conditions with zero contrast (as the same stimulus is presented continuously for two seconds).

Figure 3b shows raster plots of an example RGC’s responses to four pairs of stimuli. Again, responses were difficult to discriminate from spontaneous firing in control conditions (Fig. 3b, top row). After spontaneous activity blockade (Fig. 3b, bottom row), it was easy to see that the cell’s firing rate increased whenever it saw a positive contrast step and decreased whenever it saw a negative contrast step.

For each responsive cell in each retina and each stimulus pair, we calculated the percentage change in firing. As we were interested in how well ChR2 RGCs signal changes in irradiance against different background illuminations, we took the baseline epoch as the 500 ms before the change (to avoid the initial transient response to the first stimulus and allow each RGC’s firing to reach a steady background firing rate) and the response epoch as the 500 ms after the change in stimulus. The result is shown as a function of contrast in control conditions and after application of MFA in Fig. 3c.

There is a clear monotonic relationship between contrast and change in firing. For the purposes of this figure, we stretched out negative values on the *x* axis by defining contrast as the change in irradiance divided by the minimum irradiance of the full-field pair. If the Weber contrast (change in irradiance divided by initial irradiance) is used instead, this relationship is roughly linear (data not shown). The percentage change in firing was significantly affected by the irradiance of the first stimulus, the Weber contrast, application of MFA, and all of their two-way interactions, but not the three-way interaction (Table 1). In particular, applying MFA increased the magnitude of the change in firing, i.e. the change in firing was more positive following an increase in irradiance and more negative following a decrease in irradiance, compared to control conditions. We obtained essentially the same results using a variety of combinations of baseline and response periods, including 250 ms before and after the change, the first 250 ms of the response to each stimulus of the pair, and the whole of the response to each stimulus (Supplementary Fig. S3).

### Blockade of Spontaneous Activity Improves Spatial Acuity

**Figure 4:**
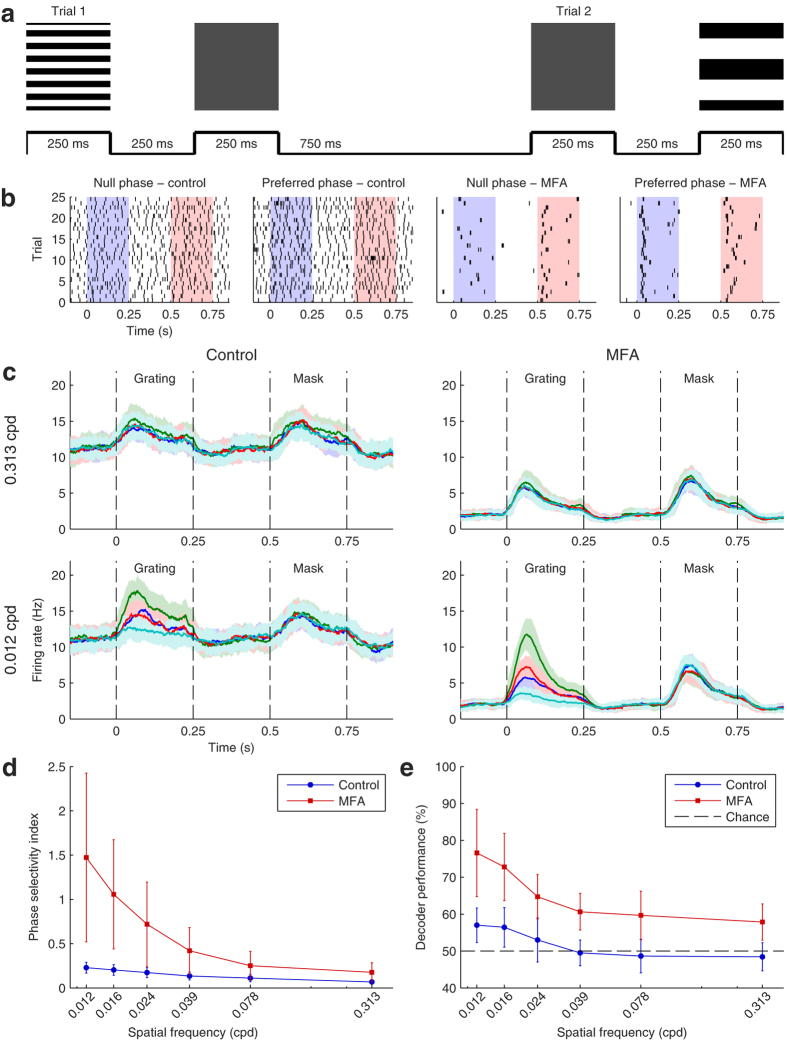
Responses of *ChR2rd1* RGCs to gratings of varying spatial frequency. **(a)** Stimulation protocol. On each trial, either a grating or an isoluminant mask was presented for 250 ms, followed by a 250 ms gap, then the mask is presented if the grating was presented first and vice versa. There is 750 ms between trials. Two example trials are shown: the top row shows the image presented to the retina during the corresponding raised portions of the timeline below. Each stimulus covers the entire MEA. **(b)** Raster plots of an example cell responding to its preferred phase of the largest grating and the opposite (null) phase in control conditions (left) and after applying 40 μM MFA (right). The blue background indicates when the grating was presented and the red background the mask. **(c)** Smoothed PSTHs averaged over all cells in an example retina, after reordering each cell’s responses so they all prefer the same phase and swapping the order of mask-first trials. Responses to the smallest (top row) and largest (bottom row) gratings are shown in control conditions (left) and after applying MFA (right). The preferred phase is shown in green and the null phase in cyan; blue and red are intermediate phases. Shaded areas represent one s.d. **(d)** Phase selectivity index (see Methods) averaged over all responsive cells in each of *n* = 6 retinas as a function of spatial frequency in control conditions (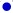) and after applying MFA (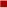). Error bars are mean ± s.d. Two-way repeated measures ANOVA showed significant main effects of spatial frequency (*F*_*GG*_(1.11, 6.64) = 19.00, *p* = 0.003), MFA (*F*(1, 5) = 12.02, *p* = 0.013), and their interaction (*F*_*GG*_(1.11, 6.68) = 12.35, *p* = 0.010). **(e)** As (**d**) for Bayesian decoder performance in distinguishing mask from grating, averaged over all grating phases in six retinas. The main effects of spatial frequency (*F*(5, 30) = 17.53, *p* = 4.1 × 10^−8^), drug (*F*(1, 5) = 46.78, *p* = 4.8 × 10^−4^), and their interaction (*F*(5, 30) = 3.61, *p* = 0.011) were all significant. The dotted black line denotes chance performance (50%).

We were also interested in how spontaneous activity affects the spatial acuity of optogenetically evoked RGC population responses. The first set of stimuli we used to investigate this was a simulated two-alternative forced choice (2AFC) task in which the goal was to distinguish gratings of varying spatial frequency and phase from an isoluminant grey full-field (mask)^27^. There were six gratings of spatial frequency varying from 0.012 to 0.313 cycles per degree (cpd) and each was presented at four different phases, for a total of 24 gratings per block. On each trial, a grating and the mask were presented for 250 ms each with a 250 ms interstimulus interval, then there was 750 ms before the next trial began (Fig. 4a). The gratings and the mask took up the entire MEA area. Between stimuli, no image was projected (i.e. the stimuli were presented against a black background). The gratings were presented in 50 randomized blocks; on half the trials the grating was presented first and on the other half the mask first. The entire stimulus protocol was then repeated after applying 40 μM MFA.

Figure 4b illustrates an example RGC’s responses to the largest grating in control conditions and after applying MFA, showing responses to the cell’s preferred and opposite (null) phases. Once again, spontaneous hyperactivity obscured the responses in control conditions, but after blocking this activity the difference in response to the preferred and null phases of the grating was clear.

Figure 4c shows smoothed, reordered, cell-average PSTHs (see Methods) for an example retina in response to the highest and lowest spatial frequency gratings in control conditions and after applying MFA. In both conditions, for the highest frequency gratings the average retinal response to the grating was similar in magnitude to the mask response and there was little separation between phases. However, for the lowest frequency gratings, there was a clear separation between the responses to the preferred and null phases, with the former receiving a much stronger response than the mask and the latter much weaker. Also of note is that the baseline firing was much lower and the difference in response with varying phase much larger after applying MFA than in control conditions. Similar results were seen in the other six retinas.

To quantify this, we calculated a phase selectivity index (PSI) for each cell (see Methods). The PSI increased as the bar size increased and applying MFA significantly increased the PSI (Fig. 4d).

Although the smoothed, reordered, cell-average PSTHs in Fig. 4c and the PSI are useful for visualization, they combine information from presentations of different stimuli to each cell. To get a better idea of how spontaneous activity affects the amount of information available on each trial, we trained a Bayesian decoder (see Methods) to decide whether the grating or the mask was presented first on each trial. The decoder was trained separately in the control and drug conditions and for each phase and frequency of grating. In each case, the training set contained responses to the relevant grating and all presentations of the mask, aside from the trial included in the test set.

Figure 4e shows the phase- and retina-average Bayesian decoder performance as a function of spatial frequency in control conditions and after applying MFA. The decoder performance was scarcely above chance at all spatial frequencies in the control condition, but performed significantly better after applying 40 μM MFA, confirming that reducing spontaneous activity improves spatial acuity.

**Figure 5:**
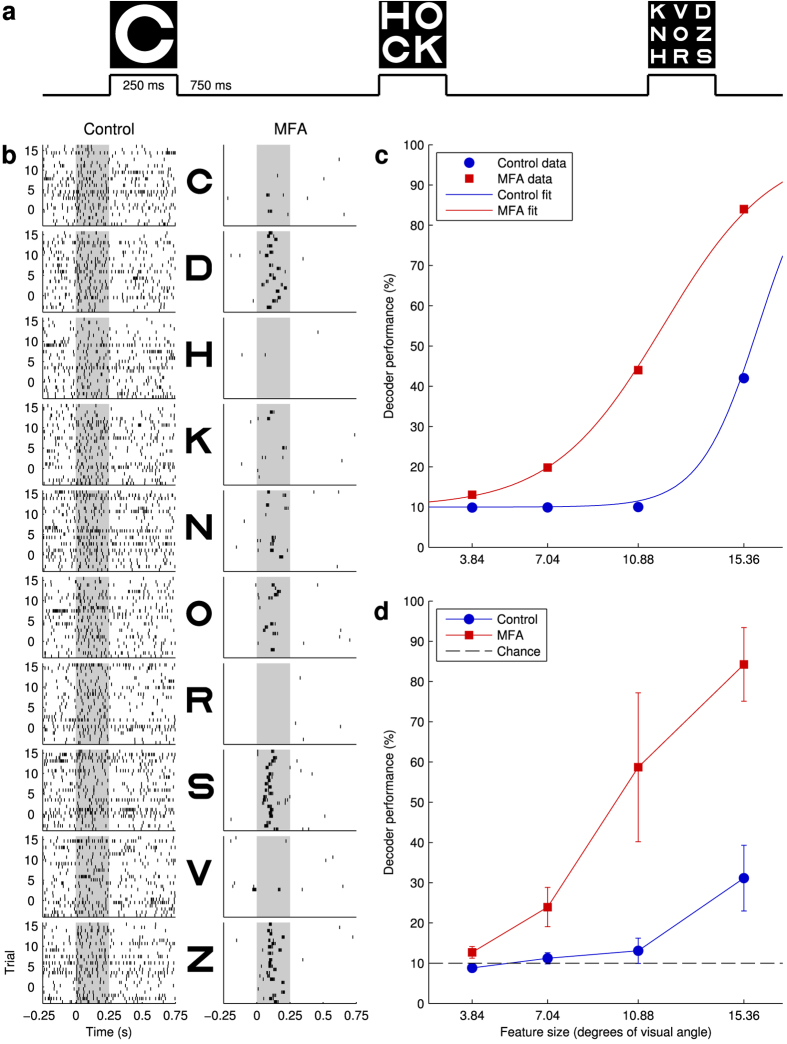
Responses of *ChR2rd1* RGCs to flashed Sloan letters. **(a)** Stimulation protocol. On each trial, a set of letters (1 each for the largest two sizes, 2 × 2 for the second smallest, and 3 × 3 for the smallest) is presented for 250 ms, followed by a gap of 750 ms. Three possible example trials are shown: the images in the top row show the image presented to the retina during the corresponding raised portion of the timeline below. **(b)** Raster plots of an example cell responding to each of the largest letters (one per row) in control conditions (left) and after applying 40 μM MFA (right). The light grey background indicates when the letter was presented. **(c)** Bayesian decoder performance in identifying letters presented on each trial (averaged over locations for the smaller letters) as a function of letter size for an example retina in control conditions (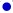) and after applying MFA (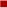). Psychometric curves fitted to the data for estimating visual acuity (see Methods) are shown as solid lines in colours matching the corresponding data points. **(d)** As (**c**), but averaged over *n* = 7 retinas and without the fitted psychometric curves. Two-way repeated measures ANOVA showed significant main effects of letter size (*F*_*GG*_(1.50, 8.98) = 128.74, *p* = 4.7 × 10^−7^) and drug (*F*(1, 6) = 75.88, *p* = 1.3 × 10^−4^), as well as a significant interaction (*F*_*GG*_(1.67, 10.03) = 48.51, *p* = 1.0 × 10^−5^). The dotted black line denotes the performance expected by chance (10%).

We also tested spatial acuity by presenting the retina with Sloan letters^28^, which are commonly used in optometry to measure patients’ visual acuity. We presented letters at four different sizes and were able to increase the intensity to ND 1.9 (1.65 mW/cm^2^), as the smaller stimuli evoke less of an artefact on the chip. For the two largest sizes, the letters covered most of the MEA and so were presented in the center of the array. For the two smallest sizes, the array was split into a 2 × 2 or 3 × 3 grid and one letter presented at each location on each trial. The letter spacing was larger than the average ChR2 RGC receptive field (~250 μm^16^), so a given cell was unlikely to be stimulated by more than one letter. 10 different letters (CDHKONRSVZ) of each size in each location were presented for 250 ms in 25 randomized blocks (Fig. 5a). All letters were presented against a black background.

Figure 5b shows raster plots of an example RGC’s responses to the 10 largest letters in control conditions and after applying MFA. It was difficult to discern any responses to any letter in control conditions (Fig. 5b, left column), but after applying MFA it became clear that this particular cell likes the D, S, and Z stimuli best, and to a lesser extent N and O. (In general, each cell will have a different set of preferred letters depending on its receptive field location.)

To quantify the amount of information about letter identity contained in the population response, for each letter size and location, we trained a Bayesian decoder to identify which letter was presented on each trial from the number of spikes fired by each RGC in the population of responsive cells. For the two largest sizes, all cells were included in the decoder. For the smaller sizes, only cells recorded on electrodes in the same grid square as the letter under consideration were used. The location-average decoder performance as a function of letter size is shown for an example retina in Fig. 5c and averaged over all retinas in Fig. 5d. The pattern is very similar to that for the gratings: in control conditions, performance was poor even for the largest letters, but after applying 40 μM MFA, the performance was significantly higher.

We used the decoder performance to estimate the visual acuity score that could be achieved by an observer using the optogenetically evoked RGC population responses to identify letters presented as on an optician’s eye chart, on the basis of the amount of information contained in the responses (on which Bayesian decoder performance places a lower bound^29^). To do this, first we fitted the decoder performance data for each retina in each condition with a sigmoid psychometric curve^30^ (see Methods; an example for one retina is shown in Fig. 5c) to allow us to estimate the decoder performance for arbitrary letter sizes. The resulting parameter estimates are shown in Table 2. Applying MFA significantly lowered the point at which the decoder achieved half-maximal performance above chance for each retina but had no significant effect on the width of the psychometric curve (which is related to the gain).

Using the fitted psychometric functions, we estimated the visual acuity of each retina before and after MFA using a simulated logMAR test (see Methods). The results of this test are also shown Table 2. The mean visual acuity was significantly higher (smaller logMAR score) after application of MFA.

### Effect of potassium concentration

**Figure 6:**
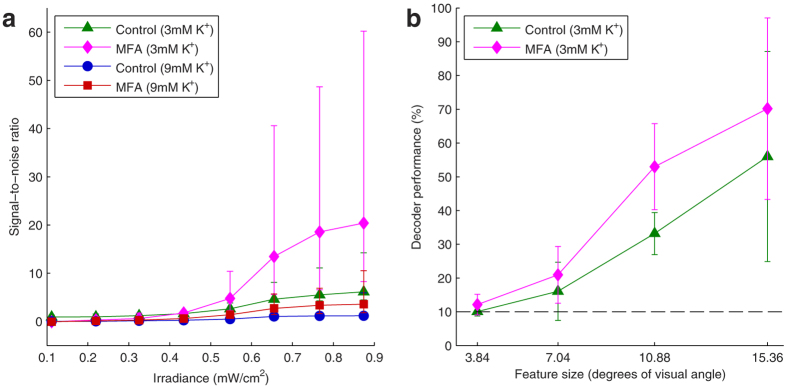
Effect of changing the extracellular potassium concentration. **(a)** Median signal-to-noise ratio for all responsive cells in one retina in which responses to full-fields of varying irradiance were recorded before adding MFA at 3 mM (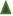) and 9 mM (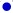) KCl and after applying 40 μM MFA at 3 mM (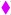) and 9 mM (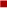) KCl. In both cases adding MFA improves the SNR, but at lower potassium concentrations the spontaneous activity is lower so the SNR is generally higher. Error bars are interquartile range. Bootstrapping revealed significant main effects of irradiance, MFA, and KCl concentration (all *p* < 0.001). **(b)** Bayesian decoder performance in identifying Sloan letters as a function of letter size averaged over *n* = 2 retinas in which responses to the Sloan letters were recorded at 3 mM KCl before (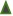) and after (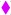) adding MFA. Error bars are mean ± s.d. Bootstrapping revealed a significant main effect of MFA (*p* = 0.036) but not feature size (p = 0.092). However, the latter may be due to the low sample size, particularly for the larger letters (one sample from each retina in each condition for these two letters sizes).

As mentioned, the above experiments were all performed at 9 mM KCl to increase the number and robustness of ChR2 responses. However, we wanted to ensure this concentration increase *per se* was not causing the improvements in visual performance, rather than MFA. Thus we conducted some control experiments in which we stimulated retinas with a subset of the stimuli used above at normal extracellular potassium levels (3 mM). In one retina, we presented full-fields of varying irradiance (as in Figs 1f and 2) before and after applying MFA at 3 mM and 9 mM KCl. Regardless of the potassium concentration, there was an increase in SNR with MFA (Fig. 6a). Moreover, the SNR was actually generally higher in the 3 mM KCl condition, most likely due to lower overall spontaneous activity in the low potassium condition (Fig 1e, Supplementary Fig. S1). In two retinas, we repeated the Sloan letters experiment at 3 mM and 9mM KCl. In both retinas, there was an improvement in decoder performance after applying MFA (Fig. 6b). Thus the light sensitivity and spatial acuity improvements demonstrated in this study are indeed due to reduction of spontaneous hyperactivity because of MFA, and not an artefact of the artificially raised potassium concentration.

## Discussion

In this study, we have demonstrated for the first time that lowering spontaneous activity in dystrophic retinas decreases the minimum irradiance and contrast necessary to evoke discernible prosthetic responses and increases the spatial frequency of features than can be successfully discriminated, as opposed to merely increasing the SNR of prosthetic responses^16^,^17^,^18^. In turn, this may extend the operating range of optogenetic retinal prostheses into lower light intensities and improve the visual acuity and experience of prosthetically restored vision for patients. Although we have used the relatively light-insensitive opsin ChR2, if these results extend to the more sensitive opsins currently being tested for retinal prosthesis^25^,^31^,^32^, this could allow an optogenetic retinal prosthesis to provide useful vision over an even greater portion of the range of light intensities encountered in daily life.

Although spontaneous activity blockade improves optogenetic responses relative to control conditions, how do the responses compare to the visual performance seen in wild-type mice and to other optogenetic retinal prosthesis designs? In the case of wild-type mice, the 2AFC grating task is similar to the task used by Jacobs *et al*.^27^ to compare retinal readout under different coding strategies to behavioral performance in the mouse. In their experiments, freely behaving mice could distinguish mask from grating perfectly up to 0.313 cycles per degree (cpd), whereas here even with MFA average performance at this spatial frequency was around 60%. However, this is comparing a mouse having two working retinas, with all their sophisticated encoding circuitry and tens of thousands of RGCs^33^, to the responses of a few hundred directly stimulated RGCs (which is typically how many responsive cells we saw in a given experiment). Moreover, their task was easier than ours in that the mouse was allowed multiple looks at a given stimulus, whereas here decoding was based on single stimulus presentations. Thus one would not expect *a priori* to recapitulate wild-type mouse visual function.

A more meaningful comparison might be with previous studies of optogenetic retinal prostheses in rodents^6^,^7^,^8^,^9^. In the first demonstration of ChR2-mediated retinal stimulation, Bi *et al*.^34^ saw weak or no activation below around 10^15^ photons·cm^−2^s^−1^. This is commensurate with intensities used in later studies^35^,^36^,^37^,^38^,^39^ (although Doroudchi *et al*.^38^ saw some improvement in visual function at 10^14^ photons·cm^−2^s^−1^). By convolving the measured relative emission spectrum of our projector with a published absorption spectrum for ChR2^40^ and multiplying by the measured retinal irradiance of 0.87 mW/cm^2^, we calculated the light from our projector at maximum brightness with a ND2.2 neutral density filter to be equivalent to a photon flux of 5.5 × 10^14^ photons·cm^−2^s^−1^ at 450 nm (the peak of ChR2’s absorption spectrum). In our experiments, we saw appreciable light responses even at the fourth brightest irradiance used (0.55 mW/cm^2^, equivalent to 3.4 × 10^14^ photons·cm^−2^s^−1^ at 450 nm; Fig. 2b–d), even at normal potassium concentrations (Fig. 6a). This is much lower than the light intensities used in previous studies, suggesting that lower spontaneous activity may significantly extend the dynamic range of optogenetic retinal prostheses and may even bring it down to realistic ambient light levels.

Similarly, various studies^35^,^41^,^42^,^43^ have investigated the ability of blind rodents expressing unmodified microbial optogenetic proteins (ChR2 or halorhodopsin) in their retinas to follow drifting gratings, as assessed using the optokinetic reflex (OKR). In general, such animals were able to follow gratings with spatial frequencies between 0.05–0.5 cpd at brightness levels on the order of 10^15^ photons⋅cm^−2^s^−1^. In this study, *ChR2rd1* RGCs were unable to distinguish gratings at similar spatial frequencies from isoluminant full-fields (decoder performance was no different than chance; Fig. 4e). If tested, these animals would have failed the optokinetic reflex test, but performance nevertheless rose above chance with MFA. The average irradiance of these contrast gratings was 0.53 mW/cm^2^, equivalent to 3.3 × 10^14^ photons⋅cm^−2^s^−1^ at 450 nm – lower than every light intensity at which OKRs were restored to genetically blind mice using algal or bacterial opsins in previous studies. Van Wyk *et al*.^25^ and Cehajic-Kapetanovic *et al*.^32^ have reported successful grating detection at lower photon fluxes, but one of these used a chimeric protein, Opto-mGluR6, that combines the light sensing domain of melanopsin with the temporal kinetics and signal amplification of the mGluR6 metabotropic glutamate receptor^25^, and the other used human rhodopsin^32^. Hence, in both of these approaches the improved light sensitivity results from signal amplification via intracellular signaling that does not take place with standard ChR2. It is possible that reducing spontaneous activity may also extend the dynamic range of these approaches, which remains to be investigated.

In summary, our results add important additional evidence to support the hypothesis that reducing spontaneous activity in dystrophic retinas improves the amount of information available in prosthetic responses to a variety of stimuli over a range of irradiances and spatial scales. This, in turn, may improve the dynamic range, contrast sensitivity and spatial acuity of optogenetically restored prosthetic vision, leading to better visual perception.

Nevertheless, many questions remain to be explored. First, we have presented a novel method for estimating retinal visual acuity using Sloan letters *in-vitro*. Although it is based on established methods for relating the information in *in-vitro* retinal responses to behavioural performance^27^ and for psychometric curve fitting^30^, it must be validated with *in-vivo* behavioural data before it can confidently be related to clinical visual acuity measures.

More generally, there are other aspects of the effect of spontaneous activity on prosthetic vision that we have yet to probe. For example, we have not probed how spontaneous activity affects temporal aspects of vision. Spontaneous hyperactivity may limit the temporal frequency of stimuli that can be perceived. Alternatively, the rhythmic, bursting nature of RGC background firing may selectively interfere with periodic stimuli of the same temporal frequency. If this is the case, these effects may be ameliorated by blocking these bursts. Additionally, although we have now tested a number of stimulus classes over a range of light intensities, contrasts, and spatial frequencies, we have yet to investigate the effect of spontaneous activity on optogenetic responses to complex natural scenes^32^,^44^.

Finally, the work presented here, as with all previous work on spontaneous hyperactivity as it relates to retinal prosthetics^16^,^17^,^18^, is based on analysis of neural population responses from electrophysiological recordings (mostly *in-vitro*, although Ivanova *et al*.^18^ also report *in-vivo* cortical data). While the results so far are encouraging, before they can be translated to the clinic they must first be validated behaviourally. Extending these results to larger animal models of RP – such as dogs^45^, cats^46^, or pigs^47^ – would also be a worthwhile step along the path to clinical translation. As well as having more sophisticated behavioural repertoires, larger mammals also have more cone-dominant driven and more regionally specialised retinas^48^. As well as being more similar to human vision, such retinas would also capitalise more on the large area and high density recording capabilities of the APS MEA.

We have discussed the challenges posed by behavioural and clinical research into pharmacological manipulation of spontaneous activity in retinal degenerations at length previously^16^. Briefly, replicating these results *in-vivo* requires a means of delivering MFA (or some other drug) to the retina in a targeted, sustained, dose-controlled manner. Also, the majority of the results presented here were achieved with higher than normal potassium concentrations, which could be toxic *in-vivo*. However, given the previous work showing the benefits of MFA and the results in Fig. 6, we do not believe it would be necessary to artificially raise the retinal potassium concentration in any attempted *in-vivo* replication of these results. Moreover, in this particular study we were restrained in the upper limit of the light intensity we can use without inducing artefactual responses on the silicon-based APS chip. Higher light intensities may provide stronger responses at normal potassium concentrations. Alternatively, a non-pharmacological approach could be employed, such as genetic cell-type specific knock-out of gap junctions^18^, gene therapy to alter AII amacrine cell potassium conductance (mimicking the effects of flupirtine^49^, but in a more targeted manner), or expressing an inhibitory optogenetic protein with a distinct absorption spectrum from ChR2 (e.g. halorhodopsin) in AII amacrine cells and silencing them with diffuse, narrowband light. Finally, we have shown that reducing spontaneous activity improves responses for an RGC-targeting, ChR2-based prosthetic. While there is no reason to suppose it would not work for other opsins as well, it is not immediately obvious that the same approach would work in a surviving cone- or bipolar cell-targeting prosthetic. This is because interfering with the inner retinal network whence the spontaneous activity originates^15^ may also disrupt the normal visual processing normally performed by that circuitry. Thus, moving forward, it will be important to determine the extent to which reducing spontaneous activity improves prosthetic responses in a variety of prosthetic designs and the best approach to block spontaneous activity for each.

## Methods

### Experimental Animals

All experimental procedures were approved by the ethics committee at Newcastle University and conducted in accordance with the UK Home Office Animals (Scientific Procedures) Act 1986. C3H/HeNHsd mice, also known as *rd1* mice (Harlan Laboratories, Indianapolis, USA) were crossbred with B6.Cg-Tg(Thy1-COP4/EYFP)9Gfng/J (*ChR2*) mice (Jackson Laboratory, Bar Harbor, USA) to generate a strain of blind mice with optogenetically sensitive RGCs (*ChR2rd1* mice).

### MEA Recordings and Optogenetic Stimulation

Detailed protocols for retinal preparation and recordings are as described elsewhere^19^ (see also Supplementary Methods). Stimuli were delivered using a custom-built projection system^50^ (see Supplementary Methods for details). We used full-fields of varying irradiance, full-field pairs of varying contrast, gratings of varying spatial frequency presented with an isoluminant full-field mask in a simulated 2AFC design^27^, and Sloan letters^28^,^51^ of varying size presented at different locations on the array. The gratings occupied the entire MEA area. Stimuli were presented in randomized blocks, such that all conditions are presented once each in random order before moving on to the next block of trials (25 blocks each for all stimuli except the full-fields in Fig. 2, for which 50 blocks were used). The projector is capable of providing a maximum irradiance on the MEA of 0.13 W/cm^2^. In most experiments, this was attenuated to 0.87 mW/cm^2^ using an ND2.2 neutral density filter to minimize light-evoked artefacts on the chip. The Sloan letters covered a smaller average area and so could be presented at higher irradiance (1.65 mW/cm^2^; ND 1.9). As the projector uses broadband RGB light, not all of the photons incident on the retina will excite ChR2 efficiently. To facilitate comparison with other optogenetic studies, a detailed account of how our irradiance measurements were converted into equivalent photon fluxes is provided in the Supplementary Methods.

### Data Analysis

All data analysis was performed with custom Matlab scripts (The MathWorks, Natick, MA, USA) except where noted. Spikes were detected from raw data using quantile-based event detection^19^,^50^,^52^ and then imported into Offline Sorter (Plexon Inc, Dallas, TX, USA) for spike sorting. Not all RGCs in a given *ChR2rd1* retina express ChR2^23^, so only cells with significant responses to test flashes interleaved with the main stimuli were included in analyses. See Supplementary Methods for more details on spike sorting and detection of responsive cells.

#### Quantifying Response Strength

The simplest measure of response strength is the mean firing rate over some response epoch, *μ*_*response*_, less the firing rate over some baseline period, *μ*_*baseline*_. We refer to this as the evoked firing rate. Precise definitions of the response and baseline epochs are given in the Results section for each set of experiments. The evoked firing rate can also be expressed as a percentage –100(*μ*_*response*_/*μ*_*baseline *_− 1) – or as a signal-to-noise ratio – (*μ*_*response*_ − *μ*_*baseline*_)/*σ*_*baseline*_, where *σ*_*baseline*_ is the standard deviation of the firing rate over the baseline period.

The percentage change in firing or the SNR can be infinite if the estimated spontaneous firing rate is zero (i.e. no spontaneous spikes are observed). For this reason, we used the median rather than the mean to estimate average firing rates for each retina. In all retinas more than half of responsive cells had at least some spontaneous firing, hence the median firing rate was finite in all cases.

#### Quantifying Grating Responses

On any given grating presentation, especially for lower spatial frequencies, each cell will see a different pattern of light in its receptive field. To visualise differences in responses to different gratings and the mask succinctly, we constructed a smoothed, reordered, cell-average PSTH as follows. First, we created a PSTH with a 1 ms bin width for each cell in each combination of grating phase, spatial frequency, and presentation order (grating-first versus mask-first) and smoothed each PSTH using a causal filter resembling a post-synaptic potential^53^:


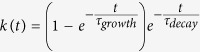


where *τ*_*growth*_ = 1 ms and *τ*_*decay*_ = 20 ms. Next, we swapped the first and second half of each mask-first PSTH. We then reordered the PSTHs within each grating frequency such that every cell had the same preferred phase (evoking the maximum firing rate). Finally, we took the cell-average PSTH for every phase, spatial frequency, and retina. Representative examples for one retina for the highest and lowest spatial frequencies in control and MFA conditions are shown in Fig. 4c.

We also created a ‘phase-selectivity index’ (PSI) for each cell equal to the average number of spikes, *P*, in response to the preferred phase for each grating less the average number of spikes, *N*, fired in response to its opposite phase, divided by the average number of spikes, *M*, fired in response to any presentation of the mask, i.e. PSI = (*P* − *N*)/*M*.

#### Bayesian Decoding

Bayesian decoding was used to quantify the amount of information available on each trial^27^,^29^ in the RGC population responses about the presentation order of the grating stimuli or the identity of the Sloan letters. Details about how we have applied Bayesian decoding to our data can be found in the Supplementary Methods.

#### Simulated LogMAR Testing

Responses to the Sloan letters were used to estimate the spatial acuity of the RGC population responses in terms of the logarithm of the minimum angle of resolution (logMAR). First, the data was fit with a psychometric curve using the psignifit Matlab toolbox (version 2.5.6)^30^ to predict the responses to arbitrary letter sizes (for details see Supplementary Methods).

Using the fitted psychometric function, we derived a visual acuity rating for each *ChR2rd1* retina before and after application of MFA using a simulated logMAR test. In a traditional logMAR test, the patient is presented with a chart of optotypes, such as Sloan letters, five to a row, where each row subtends a visual angle 0.1 decadic log units smaller than the row above (visual angles are given in minutes of arc, i.e. 1/60^th^ of a degree). The patient is then asked to read each row and given a visual acuity score 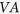 using the formula *VA* = *logMAR*_1_ + 0.1−0.02*N*, where *logMAR*_1_ is the logMAR value of the first row of letters and *N* is the total number of letters correctly identified across all rows^54^.

Normally, the largest letters on the chart subtend 10 minutes of arc^55^, i.e. a logMAR score of 1, but a *ChR2rd1* mouse is likely to have worse visual acuity than a normally sighted human. Hence, for the simulated logMAR test, we set the largest row size to 160°, i.e. a feature size of 1920 minutes of arc or a logMAR score of about 3.3. Each subsequent row was 0.1 decadic log units smaller than the previous. At each row size, the number of letters ‘read’ by each retina in each condition was estimated as five times the decoder performance (because each ‘row’ has five letters), calculated using the fitted psychometric function. The test continued until the performance dropped below one letter read, i.e. below 20%. Each retina in each condition was then assigned a visual acuity score in logMAR units using the formula above. These were converted into Snellen fractions *S* using the formula *S* = 20/(20 × 10^*VA*^).

#### Statistical Analysis

All recorded retinas were included in the relevant statistical analyses. All experimental designs were within-subjects with a single treatment group, so there was no need for randomization of or blinding towards group allocation. For all analyses presented in Figs 2, 3, 4, 5, across all levels of each factor, less than 7.5% of the samples departed significantly from normality (Shapiro-Wilk test, *p* < 0.05). In particular, none of the fitted psychometric function parameters or estimated visual acuity scores departed significantly from normality. Hence, for all comparisons involving two samples, we used paired *t*-tests to assess statistical significance, and for comparisons involving more than two samples we used repeated measures ANOVA. For the latter, where sphericity violations were detected using Mauchly’s test, we applied the Greenhouse-Geisser correction to the degrees of freedom used in the *F*-test (this is indicated in the text by writing *F*_*GG*_(*df*_1_, *df*_2_) when reporting the *F* statistic) as in all cases *ε* was less than 0.75. ANOVAs (including Mauchly’s tests and Greenhouse-Geisser corrections) were conducted in R. In Fig. 6a only one retina was present and the data were highly non-normal (in over 35% of samples the Shapiro-Wilk *p*-value was less than 0.05) and in Fig. 6b there were too few data points to assess normality, so these data were analysed by bootstrapping using the ezPerm function from the ez R package. All other statistical analyses were conducted in Matlab. The threshold for significance was set at *p* < 0.05, although all *p* values reported in the text that were significant at this level remained significant after false discovery rate correction^56^.

## Additional Information

**How to cite this article**: Barrett, J. M. *et al*. Dampening Spontaneous Activity Improves the Light Sensitivity and Spatial Acuity of Optogenetic Retinal Prosthetic Responses. *Sci. Rep.* **6**, 33565; doi: 10.1038/srep33565 (2016).

## Supplementary Material

Supplementary Information

## Figures and Tables

**Table 1:** Three-way repeated measures ANOVA table for the effect of first stimulus irradiance, Weber contrast, and application of MFA on percentage change in firing between first and second stimuli for full-field pairs.

	*DF*	*SS*	*MS*	*F*	*p*
Initial irradiance	1	82508	82508	90.34	<2 × 10^−16^*
Weber contrast	1	76915	76915	84.22	<2 × 10^−16^*
MFA	1	5648	5648	6.18	0.013*
Initial irradiance × Weber contrast	1	18150	18150	19.87	1.3 × 10^−5^*
Initial irradiance × MFA	1	16689	16689	18.27	2.8 × 10^−5^*
Contrast × MFA	1	19018	19018	20.82	8.1 × 10^−6^*
Initial irradiance × Contrast × MFA	1	1326	1326	1.45	0.23
Residuals	234	213713	913		

*DF* = degrees of freedom, *SS* = sum of squares, *MS* = mean squares. *Significant at the *p* < 0.05 level.

**Table 2:** Fitted psychometric function parameters and resulting visual acuity scores.

Retina	Control				MFA			
	*m*	*w*	*VA*	*S*	*m*	*w*	*VA*	*S*
1	15.98	5.51	3.03	21660	11.94	10.28	2.88	15087
2	15.91	0.92	3.00	20110	10.11	10.05	2.80	12625
3	17.77	7.78	3.07	23599	11.79	9.53	2.87	14933
4	18.59	10.97	3.09	24629	9.678	8.00	2.78	12177
5	15.76	1.94	3.01	20475	10.39	8.95	2.81	12893
6	18.20	9.52	3.08	24149	9.83	9.78	2.77	11794
7	17.16	8.81	3.04	22031	14.31	12.47	2.96	18293
Mean	17.05	6.49	3.05	22379	11.15	9.87	2.84	13972

*m* = midpoint of the curve (performance of 55% or half way between the chance performance of 10% and the maximum performance of 100%), *w* = width of the curve as it rises from 10% above chance (19%) to 90% above chance (91%), *VA* = estimated visual acuity score in logMAR units (see Methods), *S* = Snellen denominator, i.e. *S* = 20/(20 × 10^*VA*^). The midpoints were significantly lower after applying MFA (paired *t*-test: *n* = 7, mean ± s.d. control = 17.05 ± 1.18, drug = 11.15 ± 1.6, *t*(6) = −7.21, *p* = 3.5 × 10^−4^), as were the logMAR scores (mean ± s.d. control = 3.05 ± 0.03, drug = 2.84 ± 0.07, *t*(6) = −6.81, *p* = 4.9 × 10^−4^), but there was no significant effect on the width (mean ± s.d. control = 6.49 ± 3.85, drug = 9.87 ± 1.38, *t*(6) = 2.16, *p* = 0.073).
