# Supplementary material for: Dampening Spontaneous Activity Improves the Light Sensitivity and Spatial Acuity of Optogenetic Retinal Prosthetic Responses
Source: Sci Rep. 2016 Sep 21;6:33565. doi: 10.1038/srep33565 (PMC5030712; doi:10.1038/srep33565)
Supplement: Supplementary Information [file srep33565-s1.pdf]

Dampening Spontaneous Activity Improves the Light Sensitivity and Spatial Acuity of Optogenetic Retinal Prosthetic Responses

JOHN MARTIN BARRETT[1,2*], GERRIT HILGEN[1,2], EVELYNE SERNAGOR[1]

1. Institute of Neuroscience, Faculty of Medical Sciences, Newcastle University, Framlington Place, Newcastle-upon-Tyne, United Kingdom, NE2 4HH
2. These authors contributed equally to this work

*Corresponding Author: john.barrett@cantab.net

SUPPLEMENARY INFORMATION

SUPPLEMENTARY METHODS

MEA Recordings and Optogenetic Stimulation

Mice were killed by cervical dislocation and their eyes quickly enucleated and placed into room-temperature artificial cerebrospinal fluid (aCSF) containing (in mM) 118 NaCl, 25 $NaHCO_3$, 1 NaH_2PO_4, 3 KCl, 1 $MgCl_2$, 2 $CaCl_2$, and 10 glucose, equilibrated with 95% O_2 and 5% CO_2 for retinal dissection. The isolated retina was placed wholemount, RGC layer facing down, onto a 4096-channel CMOS MEA with platinum electrode contacts (3Brain GmbH, Landquart, Switzerland). A small piece of polyester membrane filter with 5 µm pores (Sterlitech, Kent, WA, USA) and a ring-shaped metal weight (Warner Instruments, Hamden, CT, USA) were placed on the retina to improve coupling between the tissue and the electrodes. Once in the MEA chamber, the retina was kept at 32°C and continuously perfused with aCSF at 1–2 ml/min. The retina was allowed to settle for 2 h before any recordings were taken. Electrophysiological activity was acquired at 7.1 kHz sampling rate using the Biocam and BrainWave software (3Brain GmbH, Landquart, Switzerland).

The acquisition system in our lab is equipped with a lightCrafter DLP projector (Texas Instruments, Dallas, TX, USA) and custom optomechanical setup to facilitate focusing the light from the projector onto the MEA from above[50]. This projector system provides 664x664 pixels of spatiotemporally patterned stimulation over the area of the MEA with 4 µm spatial resolution, a refresh rate of 60 Hz and full, independent control over the brightness and colour of each pixel. Custom software was used to control the stimuli and synchronize them with recordings.

As the projector uses broadband RGB light, not all of the photons incident on the retina will excite ChR2 efficiently. To facilitate comparison with other optogenetic studies, we converted these irradiance measurements into equivalent photon flux as follows. First, we measured the relative emission spectrum of the projector in the absence of any neutral density filter using a Thorlabs CCS100 Spectrometer (Thorlabs GmbH, Dachau/Munich, Germany). Using the measured spectrum and the Planck-Einstein relation, we calculated the number of photons of each wavelength necessary to achieve a given irradiance (e.g. 0.87 mW/cm^2 for ND2.2; note that this assumes that the transmission spectra of air and the neutral density filters used are reasonably flat in the visible range). Finally, we convolved this spectral photon flux density with a published absorption spectrum for ChR2[40], normalised to have a value of unity at its peak, and integrated the result to calculate the photon flux at 450 nm that would achieve the same amount of ChR2 stimulation. This value was 5.5×10^{14} $photons \cdot cm^{-2}s^{-1}$ for ND2.2 (0.87 mW/cm^2, used for most stimuli presented here) or 1.0×10^{15} $photons \cdot cm^{-2}s^{-1}$ for ND1.9 (1.65 mW/cm^2, used for the Sloan letters).

Stimuli

This section describes in detail the stimuli used in each experiment and the motivation behind each set of stimuli and their parameters. In general, all stimuli apart from the letters occupied the entire MEA array and were corrected for the nonlinear mapping between pixel values and emitted luminous flux ('gamma corrected') where appropriate. We chose to use fairly slow, static stimuli (shortest presentation time 250 ms). This was a deliberate choice, as the irradiance on the retina in our experiments is low compared to what is typically used to stimulate ChR2 and there is a trade-off between irradiance and photocurrent onset latency for ChR2[57], hence we did not attempt to probe the effect of MFA on the temporal performance of ChR2 RGC responses and used long stimuli to allow even sluggish, weakly-stimulated cells time to respond. The particular duration of 250ms was chosen on the basis of preliminary experiments not described here.

Full-fields

Full-fields occupied the entire MEA and were generated by setting all projector pixels to the same value. Where multiple grey levels were used within a given experiment, these were gamma corrected and chosen to linearly span the entire output range of the projector. For the experiments in Fig. 1f, 2, and 6a, we used a duration of 250 ms for the reasons described above. For the contrast experiments in Fig. 3, we were interested in how well *ChR2rd1* retinas signal changes in irradiance after adapting to different background light intensities, so we presented full-fields for a full second to allow each RGC time to reach a plateau firing rate before changing the light intensity.

Gratings

The grating stimuli were inspired by the task used by Jacobs et al.[27] to compare Bayesian decoding of *in-vitro* retinal responses to behavioural performance, although we used square wave instead of sine wave gratings to make the task somewhat easier. The 250 ms presentation time we used for the other stimuli is similar to their 300 ms presentation time, although we doubled the stimulus presentation frequency mainly to reduce the length of the experiments and keep the size of the data files manageable. We also alternated short (250 ms) and long (750 ms) interstimulus intervals in order to create pairs of stimuli in time and thus evaluate single trial classification performance. All gratings occupied the entire MEA area. We chose a spatial frequency of 0.313 cpd for the smallest bars: this was roughly the spatial frequency at which the behavioural performance of wild-type mouse started to deteriorate in their study. The largest bars were chosen so that one bar covered roughly half the MEA, and the frequencies of the remaining bars were spaced roughly evenly (on a logarithmic scale) between these two extremes (see Fig 4d,e for exact spatial frequencies).

Letters

The letters used were the ten Sloan letters used as optotypes in clinical visual acuity testing[28]. The 250 ms presentation time was chosen to match the experiments in Figs 2 and 4. The largest letters were chosen to cover roughly the entire array. In a preliminary experiment we used letters roughly 1/4, 1/9, and 1/16 the largest sizes, but performance bottomed out with the 1/9th scale letters, so for the experiments shown in Fig. 5 we made these letters the smallest and chose two intermediate sizes instead (features sizes of each set of letters are reported in Fig 5c,d: the feature size is one-fifth the height of the letter). To maximise coverage for the smallest two sets of letters, we split the MEA into grids and presented different letters in each grid square, analysing the data from electrodes in the different grid squares separately.

Data analysis

Spike sorting

Spikes were sorted automatically using T-distribution Expectation Maximisation[58]. Channels with more than one automatically detected unit were manually inspected to ensure correct sorting. Due to the high electrode density, the same unit was sometimes detected on multiple neighboring channels. These redundant units were removed using a custom Python script by grouping units on neighboring electrodes with greater than 5% of spikes occurring within 2 samples (~0.3ms) of another unit's spikes, then iteratively removing units from each group until only the group with the highest spike count remains.

Detecting Responsive Cells

For the experiments in Figs 2-6, certain trials were set aside for detecting responsive cells. On these trials, either no stimulus (blank trials) or a 250 ms white full-field (flash trials) was presented (one each per block). Using separate stimuli for this purpose avoids circular analysis or 'double-dipping', which increases the type I error rate[59]. To detect responsive cells, we tested whether each cell had significantly higher firing on the flash trials than the blank trials using a bootstrap test, as follows. For each cell in each recording, we constructed a PSTH of its responses on the flash trials with a bin width of 50 ms, and used the peak of the first five bins (i.e. those when the stimulus was on) to assess responsiveness. If the mean spike count in this bin was less than one per trial, the cell was excluded immediately. For each remaining cell, we divided its responses on the blank trials into 50ms bins, randomly selected a number of bins equal to the number of trials, and calculated the mean number of spikes in these bins. This procedure was repeated 10000 times to bootstrap the expected distribution of values in each bin of the flash PSTH under the null hypothesis that the cell does not respond to the flash. Using this distribution, each cell was assigned a right-tailed *p*-value

based on the peak of its flash PSTH. Cells with a Bonferroni-corrected *p*-value less than 0.05 were selected as responsive. For the experiments in Fig 1, a similar method was used, except that the firing rate during the last 500 ms of each stimulus was used to bootstrap the baseline activity distribution for the other stimulus. This amounts to asking if there is a significant change in firing with the change in stimulus. Additionally, for these experiments, the two-tailed *p*-value was used, to allow significant decreases in firing to be detected as responses. To avoid circularity in analysis of these experiments, half the trials were used for detecting responsive cells and half were used to quantify response strength.

Bayesian decoding

Bayesian decoding uses Bayes' rule to determine how likely a given stimulus *s* is to have been evoked by a response *r*:

$$p(s|r) = \frac{p(r|s)p(s)}{p(r)}$$

This approach was used in two ways. For the frequency gratings, which were presented in a simulated 2AFC task paradigm, we asked how well an animal could distinguish a grating of a given spatial frequency from an isoluminant mask using the information contained in the RGC population response by testing whether

$$p(\text{grating}|r_g) > p(\text{grating}|r_m)$$

where r_g is the response of the RGC population to the grating and r_m is the response to the mask[27]. For the Sloan letters, we attempted to decode which letter was presented in each location on each trial using the formula

$$\hat{s} = \underset{s}{\operatorname{argmax}}\, p(s|r) = \underset{s}{\operatorname{argmax}} \frac{p(r|s)p(s)}{p(r)} = \underset{s}{\operatorname{argmax}}\, p(r|s)$$

(Note that the last equality holds because *p*(*r*) is the same for all *s* and all stimuli were equiprobable, so *p*(*s*) is constant.) Then we estimated the decoder performance as the fraction of trials on which $\hat{s}$ was correctly decoded. This provides an estimate of how much information is contained in the RGC population response about the stimulus[29,44].

In all cases, *r* was taken as the vector of spike counts fired by all responsive cells during presentation of a given stimulus. This is justified as we stimulated RGCs directly via ChR2 and not via the normal

retinal network, so more sophisticated coding strategies[27,60-62] are unlikely. We used the naïve Bayesian assumption, i.e. that the responses of RGCs conditioned on the stimulus are statistically independent:

$$p(r|s) = \prod_{i=1}^{n} p(r_i|s)$$

where n is the number of RGCs and r_i is the response of the ith RGC. Again, this is justified by the fact we stimulated RGCs directly (assuming no gap-junction coupling between neighbouring ChR2-sensitive RGCs), but has been shown to work reasonably well in wild-type retinas as well[63] (but see also Schwartz et al.[62]).

When using Bayesian decoding, some portion of the trials must be used as training data to estimate $p(r|s)$ and the remainder are used for testing. We used all-but-one cross-validation, in which one trial is used as the test set and all the others are used for training; this is then repeated for the next trial until each trial has been the test trial exactly once. This has the advantage that it uses the maximum amount of training data in a given sample and allows us to ask how much information is available in the RGC population response to a single stimulus presentation; however, it does not allow estimation of the variance via different instantiations of training and test set, but reduces the performance to a single data point per retina per condition.

Psychometric curve fitting using pisgnifit

A psychometric function $\Psi(x;\mathbf{\Theta})$ gives the performance of a subject in some task as a function of some property x of the stimulus. Here, the 'subject' is the Bayesian classifier for each retina in each condition, the task is identification of the letter presented from the RGC population response, and x is the feature size of the letters. $\mathbf{\Theta} = \{m,w,\lambda,\gamma,\alpha\}$ is a parameter vector that controls the shape of the function. The fitted curves have the form:

$$\Psi(x;m,w,\lambda,\gamma,\alpha) = \gamma + (1-\gamma-\lambda)F(x;m,w,\alpha)$$

$$F(x;m,w,\alpha) = \left(1 + e^{-\frac{z(\alpha)}{w}(x-m)}\right)^{-1}$$

$$z(\alpha) = 2\log\left(\frac{1}{\alpha} - 1\right)$$

$F(x)$ is an alternative parameterization of the logistic function commonly used in generalized linear modeling and spans the range [0,1], hence $\Psi(x)$ goes from γ to $(1-\gamma-\lambda)$. γ corresponds to the 'guess

rate', i.e. the performance one would expect by chance, and is thus fixed at 10% for an 10-alternative forced choice (10AFC) task such as this. λ controls the maximum performance; however, due to the low number of data points available for fitting we decided to constrain it at zero. m gives the midpoint of the psychometric curve, i.e. the point where performance is halfway between the minimum and maximum (approximately 55% for a 10AFC task with small λ). w gives the width of the sigmoid as $F(x;m,w,\alpha)$ rises from α to $1-\alpha$, e.g. from 10% to 90% of the maximum performance above chance if $\alpha = 0.1$. Thus, small values for w correspond to a very steep (i.e. high gain) sigmoid and large w to a shallow (low gain) sigmoid. As γ and λ are fixed and the choice of α is arbitrary (0.1 is typical), this leaves the parameters to be estimated as $\mathbf{\Theta} = \{m,w\}$.

psignifit uses maximum likelihood estimation via the Nelder-Mead simplex algorithm to fit the parameters[30,64]. Additionally, psignifit allows the user to weight the likelihood function by specifying a prior distribution that expresses their belief about how plausible certain parameter values are in the absence of any data. In this study, m and w are expressed in units of degrees of visual angle, thus values less than zero or greater than 360 are nonsensical. For the width of the sigmoid, w, there is no reason *a priori* that any range of angles is more likely than any other, so we set the prior for w in this case to the uniform distribution on the interval [0,360]. For the midpoint, m, we reasoned that the minimum performance must be reached for infinitesimally small letters (0º) or larger and the maximum for letters that subtend the entire mouse retina (approximately 160º[65]) or smaller. Hence the midpoint of the psychometric curve must lie somewhere between these two extremes. Since the size of the features used to distinguish the letters is one-fifth the size of the letters themselves[28], this corresponds to the range 0-32º. Thus, for m we used a Gaussian prior with mean 16 and standard deviation 8, which assigns low probability to values less than 0º or more than 32º and high probability to values in the middle. Additionally, we reran the analysis with a number of other priors, but the conclusions were largely the same even if the exact fitted parameter values varied.

SUPPLEMENTARY FIGURES

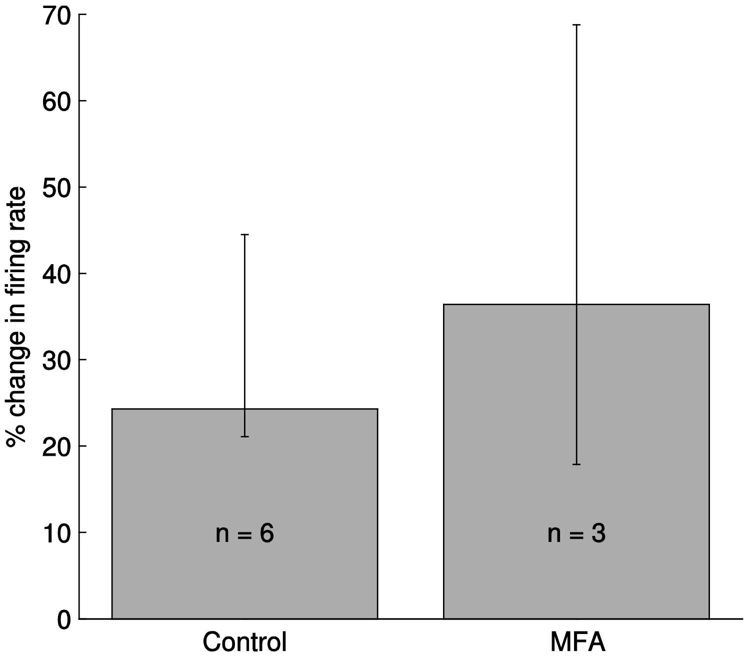

Figure S1: change in spontaneous firing with increasing potassium. This is the same data from Fig. 1e replotted as a percentage change in spontaneous firing between the 3 mM and 9 mM KCl conditions. *n* numbers represent the number of retinas in each condition. Comparisons were made within cells, hence not all retinas could be included in all conditions.

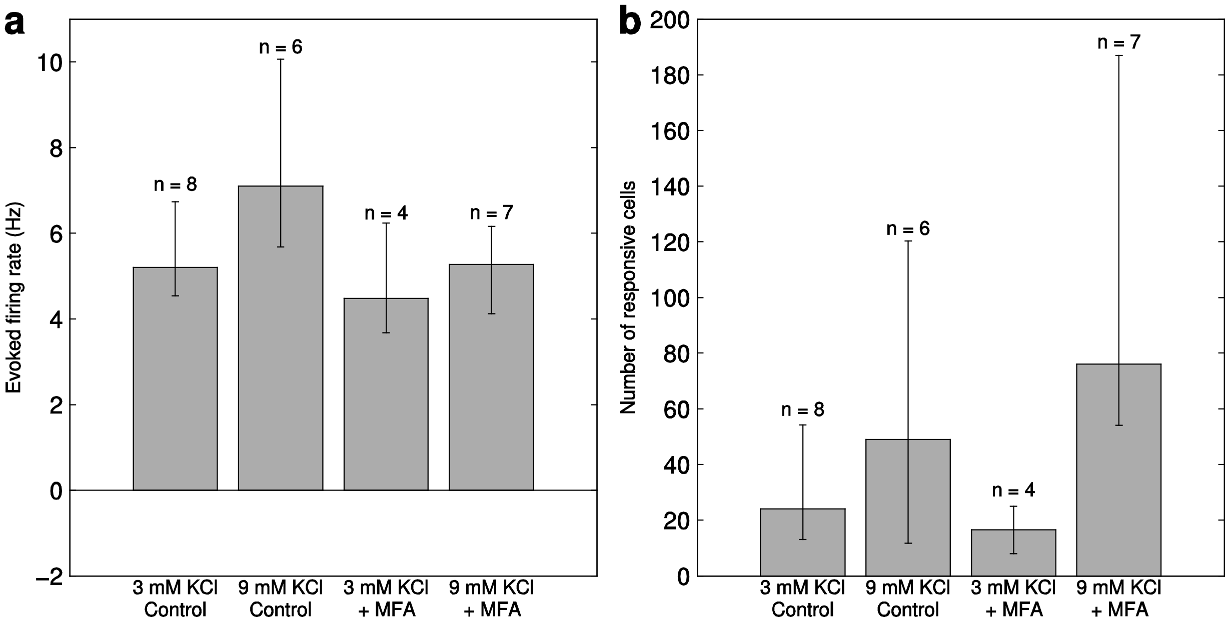

Figure S2: ChR2 responsiveness versus MFA and KCl concentration. **(a)** Median evoked firing rate during the first 250 ms in response to full-field white flashes (these data are pooled from several recordings where either 250 ms or 2 s duration flashes were presented) in the presence of either 3 or 9 mM KCl and 0 or 40 µM MFA. *n* numbers represent the number of distinct retinas recorded from in each condition. Medians are taken first over all responsive cells, then over retinas. Error bars are interquartile range. **(b)** Median number of responsive cells in each condition.

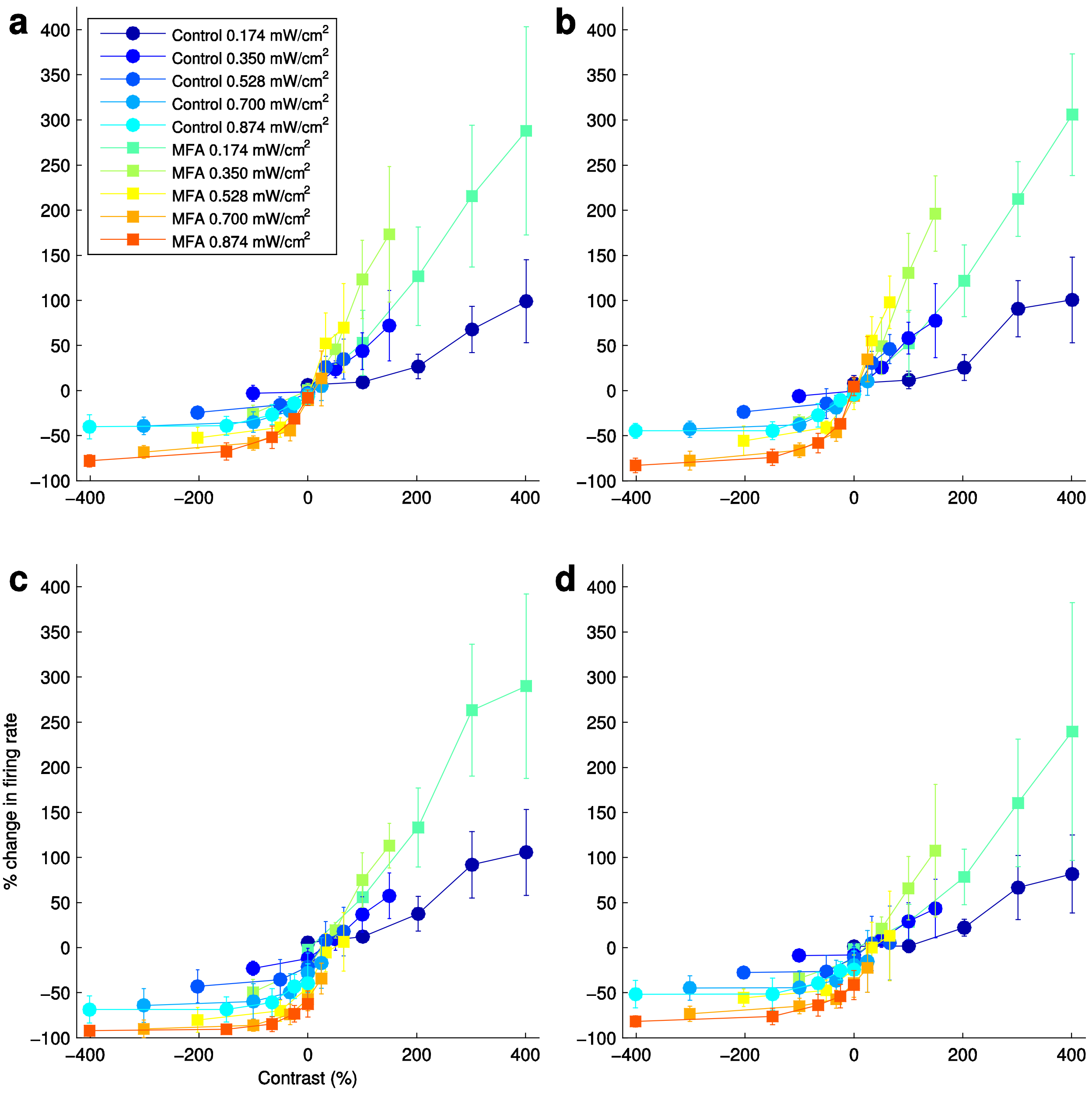

Figure S3: contrast responses using different definitions of baseline and response periods. (a) The data from Fig. 3c. **(b)** The same analysis using the 250 ms immediately prior to the change in luminance as the baseline and the 250 ms after as the response. **(c)** The same analysis using the first 250 ms of the first stimulus as the baseline and the first 250 ms of the second as the response. **(d)** The same analysis using the whole duration of the first stimulus as the baseline and the whole duration of the second as the response.

References

57. Lin, J. Y., Lin, M., Steinbach, P. & Tsien, R. Characterization of engineered channelrhodopsin variants with improved properties and kinetics. *Biophys. J.* **96,** 1803–1814 (2009).

58. Shoham, S., Fellows, M. R. & Normann, R. A. Robust, automatic spike sorting using mixtures of multivariate t-distributions. *J. Neurosci. Methods* **127,** 111–112 (2003).

59. Kriegeskorte, N., Simmons, W. K., Bellgowan, P. S. F. & Baker, C. I. Circular analysis in systems neuroscience: the dangers of double dipping. *Nat. Neurosci.* **12,** 535–40 (2009).

60. Gollisch, T. & Meister, M. Rapid neural coding in the retina with relative spike latencies. *Science (80-.).* **319,** 1108–1111 (2008).

61. Van Rullen, R. & Thorpe, S. J. Rate coding versus temporal order coding: What the retinal ganglion cells tell the visual cortex. *Neural Comput.* **13,** 1255–1283 (2001).

62. Schwartz, G., Macke, J., Amodei, D., Tang, H. & Berry, M. J. Low error discrimination using a correlated population code. *J. Neurophysiol.* **108,** 1069–88 (2012).

63. Nirenberg, S., Carcieri, S. M., Jacobs, A. L. & Latham, P. E. Retinal ganglion cells act largely as independent encoders. *Nature* **411,** 698–701 (2001).

64. Press, W. H., Teukolsky, S. A., Vetterling, W. T. & Flannery, D. P. Minimization or maximization of functions in *Numerical Recipes: The Art of Scientific Computing* 487–562 (Cambridge University Press, 2007).

65. Marc, R. E. Schematic mouse, rat and human eyes. *MarcLab* http://prometheus.med.utah.edu/~marclab/eyes.pdf (2006)
